# Inhibition of p90 ribosomal S6 kinases disrupts melanoma cell growth and immune evasion

**DOI:** 10.1186/s13046-023-02755-5

**Published:** 2023-07-19

**Authors:** Corinna Kosnopfel, Simone Wendlinger, Heike Niessner, Johannes Siewert, Tobias Sinnberg, Angelika Hofmann, Jonas Wohlfarth, David Schrama, Marion Berthold, Claudia Siedel, Birgit Sauer, Aarthi Jayanthan, Georg Lenz, Sandra E. Dunn, Bastian Schilling, Birgit Schittek

**Affiliations:** 1 Department of Hematology, Oncology and Pneumology, University Hospital Muenster, 48149 Muenster, Germany; 2 Department of Dermatology, Venereology and Allergology, University Hospital Wuerzburg, 97080 Wuerzburg, Germany; 3 Mildred Scheel Early Career Center Wuerzburg, University Hospital Wuerzburg, 97080 Wuerzburg, Germany; 4 Division of Dermatooncology, Department of Dermatology, University of Tuebingen, 72076 Tuebingen, Germany; 5 Department of Dermatology, Venereology and Allergology, Charité-Universitätsmedizin Berlin, 10117 Berlin, Germany; 6 Phoenix Molecular Designs, Vancouver, BC Canada

**Keywords:** Melanoma, p90 ribosomal S6 kinase, Tumor growth, Melanocyte differentiation antigens, Immunogenicity

## Abstract

**Background:**

The mitogen-activated protein kinase (MAPK) signaling pathway is frequently hyperactivated in malignant melanoma and its inhibition has proved to be an efficient treatment option for cases harboring BRAF^V600^ mutations (BRAF^Mut^). However, there is still a significant need for effective targeted therapies for patients with other melanoma subgroups characterized by constitutive MAPK activation, such as tumors with NRAS or NF-1 alterations (NRAS^Mut^, NF-1^LOF^), as well as for patients with MAPK pathway inhibitor-resistant BRAF^Mut^ melanomas, which commonly exhibit a reactivation of this pathway. p90 ribosomal S6 kinases (RSKs) represent central effectors of MAPK signaling, regulating cell cycle progression and survival.

**Methods:**

RSK activity and the functional effects of its inhibition by specific small molecule inhibitors were investigated in established melanoma cell lines and patient-derived short-term cultures from different MAPK pathway-hyperactivated genomic subgroups (NRAS^Mut^, BRAF^Mut^, NF-1^LOF^). Real-time qPCR, immunoblots and flow cytometric cell surface staining were used to explore the molecular changes following RSK inhibition. The effect on melanoma cell growth was evaluated by various two- and three-dimensional in vitro assays as well as with melanoma xenograft mouse models. Co-cultures with gp100- or Melan-A-specific cytotoxic T cells were used to assess immunogenicity of melanoma cells and associated T-cell responses.

**Results:**

In line with elevated activity of the MAPK/RSK signaling axis, growth and survival of not only BRAF^Mut^ but also NRAS^Mut^ and NF-1^LOF^ melanoma cells were significantly impaired by RSK inhibitors. Intriguingly, RSK inhibition was particularly effective in three-dimensional growth settings with long-term chronic drug exposure and suppressed tumor cell growth of in vivo melanoma models. Additionally, our study revealed that RSK inhibition simultaneously promoted differentiation and immunogenicity of the tumor cells leading to enhanced T-cell activation and melanoma cell killing.

**Conclusions:**

Collectively, RSK inhibitors exhibited both multi-layered anti-tumor efficacy and broad applicability across different genomic melanoma subgroups. RSK inhibition may therefore represent a promising novel therapeutic strategy for malignant melanoma with hyperactivated MAPK signaling.

**Supplementary Information:**

The online version contains supplementary material available at 10.1186/s13046-023-02755-5.

## Background

The mitogen-activated protein kinase (MAPK) signaling pathway plays a crucial role in melanocyte biology, regulating both proliferation and melanogenesis [1]. Consequently, it is not surprising that the MAPK signaling cascade is frequently constitutively activated by mutations in cutaneous melanoma and represents a key driver of melanoma progression and invasion [2, 3]. The Cancer Genome Atlas (TCGA) program conducted a systematic, multi-dimensional characterization of a panel of cutaneous melanomas underscoring the pivotal role of MAPK signaling with a high prevalence and often mutual exclusivity of specific mutations in distinct genes of this pathway [4, 5]. Based on that, a genomic classification of cutaneous melanoma into four subtypes is proposed: i) BRAF subtype (~ 50% of cutaneous melanomas; activating BRAF mutations; BRAF^Mut^), ii) RAS subtype (20–30%; activating mutations in the RAS isoforms NRAS, HRAS and KRAS; RAS^Mut^), iii) NF-1 subtype (10–15%; loss-of-function mutations of NF-1; NF-1^LOF^) and iv) triple wild-type (WT) subtype (10–15%; absence of NF-1, BRAF or RAS mutations).

The discovery of small molecule inhibitors selective for mutated BRAF marked the beginning of a new era in melanoma therapy, exposing the MAPK signaling pathway as a very promising therapeutic target [3, 6]. The application of BRAF inhibitors, first as monotherapy and subsequently also in combination with MEK inhibitors, significantly improved response rates and prolonged progression-free and overall survival of patients with BRAF^Mut^ melanomas [7, 8]. However, its clinical benefit is limited by an almost inevitable and in part rapidly emerging resistance, which often goes along with a reactivation of MAPK signaling [9–11]. Moreover, the development of potent targeted therapies for other MAPK pathway-hyperactivated genomic subtypes, the RAS^Mut^ and NF-1^LOF^ subtypes, still represents a substantial unmet medical need [12, 13]. This is of particular concern given that immunotherapeutic regimens, which represent an effective alternative treatment option for malignant melanoma patients, induce durable response in only approximately half of all cases [14, 15].

The p90 ribosomal S6 kinase (RSK) protein family is a central downstream effector of active MAPK signaling, directly activated by ERK. It is involved in the regulation of key cellular processes including cell growth and proliferation as well as cell survival and invasiveness (comprehensively reviewed in Romeo et al. [16]). Accordingly, there is a long list of tumor entities exhibiting deregulated expression and/or activity of RSK family members [17]. Along with a MAPK pathway hyperactivation, BRAF-mutated melanoma cells exhibit increased RSK activity and can be effectively targeted by RSK inhibition particularly in the case of MAPK pathway inhibitor resistance [18].

Thus, this study explored a potential universal efficacy of RSK inhibitors in malignant melanoma with MAPK pathway hyperactivation – including RAS- and NF-1-mutated in addition to BRAF-mutated melanomas. To this end, the direct effect of RSK inhibition on melanoma cell growth and survival was examined using different in vitro and in vivo melanoma models. This was facilitated by PMD-026, a novel first-in-class RSK inhibitor with oral bioavailability. Moreover, a potential influence of the MAPK/RSK signaling cascade on melanoma cell differentiation and immunogenicity was investigated.

## Methods

### Cell culture

Primary melanocytes as well as patient-derived melanoma cells (TüMel, UKW-Mel) were isolated from human tissue (4 for primary melanocytes, 5 for patient-derived melanoma cells) and cultured as previously described [19, 20]. The use of human tissue was approved by the respective local medical ethical committees (Tübingen: 43/2008B01, 16/2009B02, 40/2009B02; Würzburg: 241/2014) and experiments were performed according to the Declaration of Helsinki Principles.

Melanoma cell lines (Suppl. Table S1) were cultured and regularly tested for mycoplasma contamination as previously described [21]. Cells were used no longer than two months upon thawing of the frozen stock. Melanoma cells with acquired BRAF inhibitor (vemurafenib) resistance or double resistance to BRAF and MEK inhibitors (cobimetinib) were generated by cultivation with increasing concentrations of the respective inhibitors over several months (final concentrations: 2 µM vemurafenib, 200 nM cobimetinib). Inhibitor-free cell culture medium was used 24 h before the resistant cells were subjected to experiments.

### Melanoma and T cell co-cultures

For generation of cytotoxic T cells with gp100- or Melan-A-specific human leukocyte antigen A*02 (HLA-A*02)-restricted T-cell receptors (TCRs), peripheral blood mononuclear cells (PBMCs) from healthy donors were isolated using Biocoll separating solution (Merck; Darmstadt, Germany). PBMCs were washed twice with phosphate buffered saline (PBS) and CD8^+^ T cells were magnetically separated with CD8-MicroBeads using an autoMACS^®^ Pro Separator (both Miltenyi Biotec; Bergisch Gladbach, Germany). Resulting fractions were analyzed for purity with CD45- and CD8-specific antibodies via flow cytometry. CD8^+^ fractions always consisted of  > 95% CD8^+^ T cells.

Lentiviral particles were produced in HEK293T cells as previously described [22] using a third-generation packaging system (pRSV-Rev, pMD2.G and pMDLg/pRRE) and lentiviral transfer vectors encoding for gp100- or Melan-A-specific TCRs (pcDH_gp100_TCR_puro_EGFP: targets gp100 peptide 154–162 on HLA-A*02; pcDH_Melan-A_TCR_puro_EGFP: targets Melan-A peptide 27–35 on HLA-A*02).

Freshly separated CD8^+^ T cells were incubated with CD3/CD28 Dynabeads (Thermo Fisher Scientific; Waltham, MA, USA) 24 h before spinoculation (45 min at 800xg) with lentivirus containing supernatants supplemented with 5 µg/mL Polybrene and 10 µg/mL Synperonic F108 (both Merck; Darmstadt Germany). After transduction, medium was changed every second day with addition of 50 U/mL interleukin-2. On the sixth day (d) after separation, cells were analyzed for expression of the transduced TCR by flow cytometry using TCR-specific major histocompatibility complex (MHC) tetramers (Flex-T™ HLA-A*02:01 Monomer UVX, loaded with gp100 or Melan-A peptide and tetramerized with allophycocyanin (APC)-Streptavidin; BioLegend; San Diego, CA, USA). On day ten, the transgenic T cells were co-cultured with HLA-A*02-positive melanoma cells, which have been pre-treated with the indicated inhibitors for 72 h. Co-cultures were performed in polypropylene tubes at a 1:1 ratio (75,000 cells each in 1 mL culture medium) for 24 h.

### Cell viability assays

Viability of cells grown in monolayer cultures was assessed using the AlamarBlue assay as described previously [23].

The viability of melanoma cells after co-culture with T cells was determined by staining with 7-Amino-Actinomycin (7-AAD; Thermo Fisher Scientific; Waltham, MA, USA) and AnnexinV-APC (BD Biosciences; East Rutherford, NJ, USA) for 15 min at room temperature. Viable cells were defined as 7-AAD-/AnnexinV-double negative and measurements were conducted with the BD FACSCanto™ flow cytometer (BD Biosciences).

### Cell cycle analyses

Cell cycle analyses were conducted by flow cytometry as described previously [21].

### Spheroid growth assay

Melanoma spheroids were generated via the “hanging drop” method with 250 melanoma cells in 25 µL culture medium applied to the lid of a non-adhesive, PBS-filled petri dish. After 10 d, spheroids were embedded in 0.5% agar noble (BD Difco/Thermo Fisher Scientific; Waltham, MA, USA) containing culture medium on a 24-well plate. The indicated small molecule inhibitors were added to the culture medium on top of the embedded spheroids and exchanged twice a week. Microphotographs were taken every other or third day and spheroid sizes (area of spheroid cross sections) were quantified using ImageJ (Wayne Rasband, National Institutes of Health; Bethesda, MD, USA).

### Anchorage-independent growth assay

Anchorage-independent growth of melanoma cells in soft agar was assessed as reported previously [21] using culture medium supplemented with 10% fetal calf serum (FCS) and the indicated inhibitors. After 10 d, colonies were counted with a phase-contrast microscope (Olympus; Hamburg, Germany).

### In vivo xenograft growth assay

To assess melanoma growth in vivo, 1 × 10^6^ melanoma cells (NF-1^LOF^: MeWo; BRAF^Mut^: 451LU) in 100 µL PBS/Matrigel (1:1) were subcutaneously injected into the right flank of NOD *scid* gamma (NSG™; NOD.*Cg-Prkdc*^*scid*^*Il2rg*^*tm1Wjl*^*/SzJ*) mice. After formation of palpable tumors, mice were randomized into two (NF-1^LOF^, *n* = 7) or four (BRAF^Mut^, *n* = 7 or *n* = 6) treatments groups, respectively, based on gender, age, and weight. PMD-026 (100 mg/kg in 10% dimethyl sulfoxide (DMSO) and 2% CremophorEL) or vehicle only was applied twice a day by oral gavage. The BRAF^Mut^ xenograft model additionally received a chemical additive diet containing 417 parts per million (ppm) vemurafenib (LC Laboratories; Woburn, MA, USA) or standard food *ad libitum* (ssniff Spezialdiäten GmbH; Soest, Germany). Treatment (NF-1^LOF^: 14 d; BRAF^Mut^: 10 d) was prematurely stopped if tumors ulcerated or exceeded a volume of 1,000 mm^3^. Tumor size was monitored every other day by measurement of tumor length and width using a caliper and the tumor volume (V) was calculated employing the following formula: V = 0.4 × length × width^2^. All animal experiments were approved by the responsible regional authority (Regierungspräsidium Tübingen, AZ HT1/18).

### Real-time quantitative polymerase chain reaction (qPCR) analysis

Total RNA was extracted with the my-Budget RNA Mini Kit (Bio-Budget Technologies; Krefeld, Germany) and reverse transcribed into cDNA using the SuperScript II Reverse Transcriptase (Thermo Fisher Scientific; Waltham, MA, USA). Real-time qPCR analysis was performed with the Takyon™ Low ROX SYBR^®^ Master Mix dTTP Blue (Eurogentec; Seraing, Belgium) on a qTOWER^3^ G real-time thermocycler (Analytik Jena; Jena, Germany). Primer sequences are listed in Supplementary Table S2.

### Western blot

Whole-cell lysates were generated and used in Western blot analysis as described previously [21]. Lysates of flash frozen xenograft samples were generated with the NucleoSpin TriPrep Kit (Macherey-Nagel; Düren, Germany) after homogenization with a rotor-stator homogenizer (Ultra-TURRAX T25; IKA; Staufen, Germany). Primary antibodies are listed in Supplementary Table S3. Immunoreactive bands were densitometrically assessed using ImageJ (Wayne Rasband, National Institutes of Health; Bethesda, MD, USA).

### Immunohistochemistry

Immunohistochemical staining of formalin-fixed paraffin-embedded melanoma xenografts was conducted as described [23] with anti-P^S102^-YB-1 as primary antibody (1:30 dilution; Cell Signaling Technology; Leiden, The Netherlands).

### Flow cytometric cell surface staining

Surface expression of MHC class I molecules (HLA-ABC, HLA-A*02) on melanoma cells and of the activation marker CD25 on TCR-transduced T cells were quantified with a CytoFLEX flow cytometer (Beckman Coulter; Brea, CA, USA) using fluorophore-coupled antibodies: APC-HLA-A*02 (BB7.2; eBioscience/Thermo Fisher Scientific; Waltham, MA, USA), fluorescein isothiocyanate (FITC)-HLA-ABC (W6/32), APC-CD25 (BC96) (both BioLegend; San Diego, CA, USA). Data analysis was performed with FlowJo™ v10.8 Software (BD Life Sciences; East Rutherford, NJ, USA).

### ELISA

Interferon-γ (IFNγ) in culture supernatants was quantified with the ELISA MAX™ Deluxe Set Human IFN-γ Kit (BioLegend; San Diego, CA, USA) according to the manufacturer’s recommendations.

### Statistical analysis

Statistical analyses were performed as indicated using GraphPad Prism version 9.5.0 (GraphPad Software; Boston, MA, USA). *p*-values < 0.05 were considered statistically significant (* for *p* < 0.05, ** for *p* < 0.01, *** for *p* < 0.001, **** for *p* < 0.0001).

## Results

### Genomic melanoma subgroups with MAPK pathway hyperactivation exhibit active RSK signaling

**Fig. 1:**
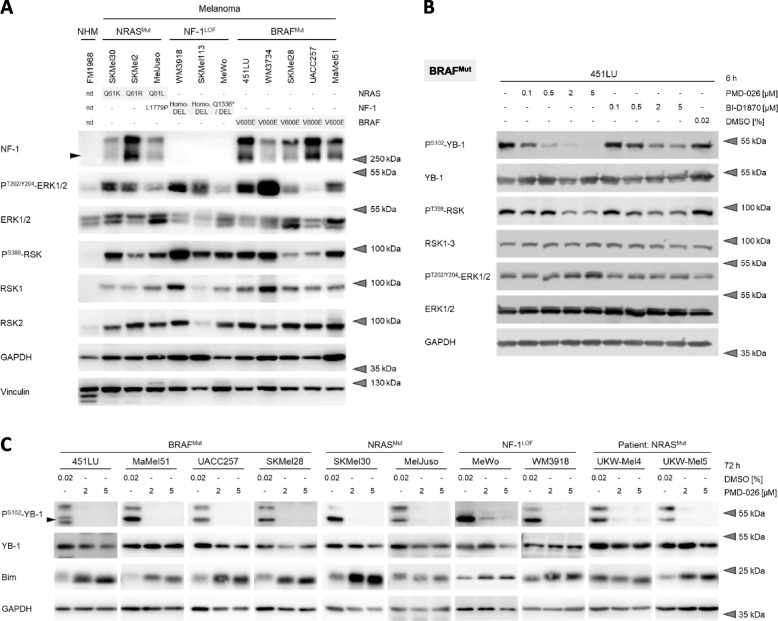
Active RSK signaling in MAPK pathway-hyperactivated melanoma cells is effectively blocked by RSK-specific inhibitors. **A** Western blot analysis of the MAPK/RSK signaling axis in whole cell protein lysates of MAPK pathway-hyperactivated melanoma cells of different genomic subgroups (NRAS^Mut^, NF-1^LOF^, BRAF^Mut^) and of primary melanocytes (NHM, normal human melanocytes) as benign control cells. GAPDH and Vinculin were used as loading controls. **B**,** C** Immunoblot analysis of RSK activity in whole cell protein lysates of 451LU (BRAF^Mut^, B) or a panel of MAPK pathway-hyperactivated melanoma cell lines (BRAF^Mut^, NRAS^Mut^, NF-1^LOF^) and patient-derived short-term cultures (C) after treatment with increasing doses of RSK inhibitors (PMD-026, BI-D1870) or solvent control treatment with DMSO for 6 h (B) or 72 h (C), respectively. GAPDH served as loading control

To investigate a potential general role of RSK in melanoma with hyperactivated MAPK signaling, we used a panel of cell lines including melanoma cells with activating NRAS mutations (NRAS^Mut^), BRAF mutations (BRAF^Mut^) or an NF-1 loss-of-function (NF-1^LOF^) (Fig. 1A; Suppl. Figure S1A). Along with an activation of the MAPK pathway reflected by ERK1/2 phosphorylation, the analyzed melanoma cell lines not only commonly expressed the oncogenic RSK family members RSK1 and RSK2 but also exhibited marked RSK activity indicated by its activating phosphorylation at serine 380 (P^S380^-RSK) and threonine 359 (P^T359^-RSK). This was independent of the genetic mechanism underlying MAPK pathway hyperactivation and in contrast to primary normal human melanocytes (NHM) as benign control cells (Fig. 1A; Suppl. Figure S1A; Suppl. Table S4).

Phosphorylation of the well-described RSK target Y-box binding protein 1 (YB-1) at serine 102 (P^S102^-YB-1), a surrogate marker for RSK activity, could be effectively and dose-dependently suppressed in 451LU and WM3918 melanoma cell lines as respective models for the BRAF^Mut^ and NF-1^LOF^ mutational subgroups. This was shown with both the established RSK-specific small molecule inhibitor BI-D1870 and the novel first-in-class RSK inhibitor PMD-026 (Fig. 1B; Suppl. Figure S1B; Suppl. Table S5). A 24-h treatment with either of the two RSK inhibitors confirmed successful pathway inhibition in a wide range of melanoma cell lines with different genetic background (Suppl. Figure S1C; Suppl. Table S6). The efficacy of RSK inhibition could be retained after 72 h and extends to patient-derived melanoma short-term cultures (UKW-Mel4, UKW-Mel5). This was demonstrated by a reduced phosphorylation not only of YB-1 but also of SRF, a second RSK target, as well as by increased levels of the pro-apoptotic protein Bim, which is known to be targeted for proteasomal degradation by RSK (Fig. 1C; Suppl. Figure S1D, E; Suppl. Table S7, S8). Of note, the novel RSK inhibitor PMD-026 appears to be more potent compared to the established inhibitor BI-D1870, leading to stronger suppression of P^S102^-YB-1 levels (Fig. 1B; Suppl. Figure S1C; Suppl. Table S5, S6) and thus could serve as a valuable tool for targeting RSK activity in malignant melanoma with constitutive activation of the MAPK pathway.

### RSK inhibition impairs viability and growth of melanoma cells with MAPK pathway hyperactivation

**Fig. 2:**
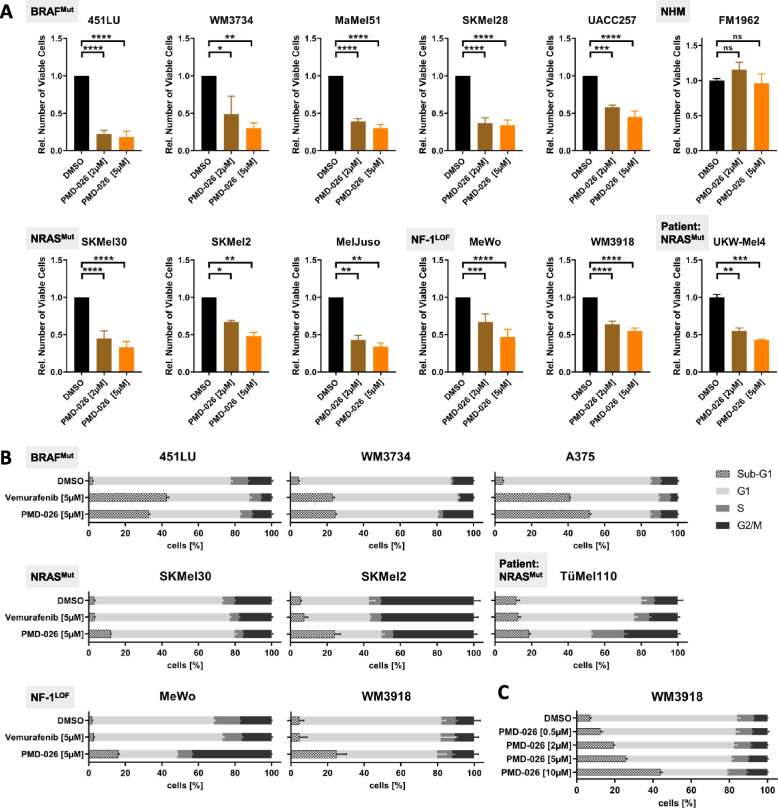
RSK inhibition negatively affects growth and survival of MAPK pathway-hyperactivated melanoma cells. **A** Cell viability (AlamarBlue assay) of melanoma cell lines from different mutational subgroups (BRAF^Mut^, NRAS^Mut^, NF-1^LOF^), patient-derived melanoma short-term cultures as well as of melanocytes (NHM) as benign control cells after treatment with the RSK inhibitor PMD-026 for 72 h (N ≥ 2 with *n* = 3; mean ± standard deviation (SD)). Viability was normalized to solvent-treated control cells and significance determined by one-way ANOVA with subsequent Tukey’s multiple comparisons test. **B**,** C** Flow cytometric cell cycle analyses of melanoma cell lines and a melanoma patient-derived short-term culture (TüMel110) following treatment with PMD-026 or the BRAF^V600E/K^ inhibitor vemurafenib for 3 d (*n* = 3; mean ± SD)

In line with the observed stabilization of the pro-apoptotic protein Bim following RSK inhibition (Fig. 1C; Suppl. Figure S1E; Suppl. Table S7), increasing doses of the RSK inhibitor PMD-026 significantly diminished the number of viable cells after a 72-h treatment throughout the BRAF^Mut^, NRAS^Mut^ and NF-1^LOF^ mutational subgroups as well as in a patient-derived short-term culture (UKW-Mel4) (Fig. 2A). In contrast, primary melanocytes as benign control cells remained unaffected (Fig. 2A).

Similarly, flow cytometric cell cycle analyses revealed that PMD-026 treatment could strongly induce sub-G1 fractions, an indicator for apoptosis, or increase the G2/M cell cycle fraction suggesting a G2/M cell cycle arrest in multiple melanoma cell lines irrespective of their mutational status (Fig. 2B; Suppl. Figure S2A). Intriguingly, PMD-026 showed a comparable efficacy in sub-G1 induction to the BRAF^V600E/K^ inhibitor vemurafenib in the BRAF^Mut^ melanoma cell lines (Fig. 2B; Suppl. Figure S2A). However, in contrast to the latter, PMD-026 retained its inhibitory function in melanoma cells with acquired resistance to the BRAF inhibitor (R) as well as to a BRAF and MEK inhibitor combination (RR) (Suppl. Figure S2B). As shown for the NF-1^LOF^ cell line WM3918, the effect of PMD-026 on the cell cycle distribution of melanoma cells was dose-dependent starting at already low drug concentrations (Fig. 2C).

**Fig. 3:**
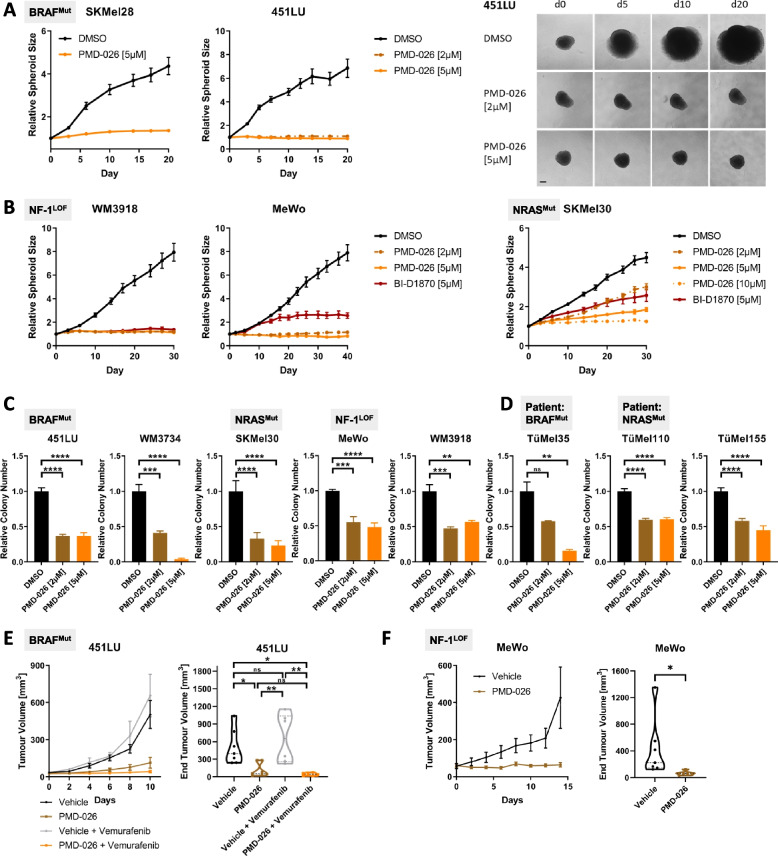
RSK inhibitors effectively impair melanoma cell growth under three-dimensional growth conditions and in vivo. **A**, **B** Growth of melanoma spheroids embedded in soft agar under RSK inhibition. Microphotographs of the spheroids were taken over the course of treatment (**A**: right panel; scale bar represents 200 µm). Spheroid sizes were quantified with ImageJ and normalized to the initial spheroid size at treatment start (mean ± standard error of the mean (SEM); A: left panel; **B**). Two independent experiments with *n* ≥ 3 were performed. **C**, **D** Anchorage-independent growth assay of melanoma cell lines (**C**) and patient-derived short-term cultures (**D**) under RSK inhibitor treatment (PMD-026). After 10 d, colonies were visualized with crystal violet, counted, and normalized to the solvent-treated controls (N = 2 with *n* = 5, mean ± SD). Significance was determined by one-way ANOVA with subsequent Tukey’s multiple comparisons test. **E**,** F** Subcutaneous xenograft growth of melanoma cells (E: BRAF^Mut^; F: NF-1^LOF^) in NOD *scid* gamma (NSG) mice under RSK inhibitor therapy. BRAF-mutated 451LU were additionally treated with the BRAF^V600E/K^ inhibitor vemurafenib and its combination with PMD-026 (**E**). Mean tumor growth (± SEM; left panel) and tumor end volumes (violin plot including individual values, quartiles and median; right panel) for the respective treatment groups are shown (E: *n*_Vehicle_, *n*_PMD-026_ = 7, *n*_Vemurafenib_, *n*_PMD-026+Vemurafenib_ = 6; F: *n* = 7). Significance was determined by one-way ANOVA with subsequent Tukey’s multiple comparisons test (E) or with a two-tailed unpaired t-test (**F**)

Corresponding to the decreased survival and/or the induction of cell cycle arrest upon RSK inhibition, we detected a strong suppression of spheroid growth throughout the mutational melanoma subgroups by PMD-026, which was confirmed by the second RSK inhibitor BI-D1870 (Fig. 3A, B; Suppl. Figure S3). Remarkably, PMD-026 completely suppressed the three-dimensional growth of most melanoma cell lines already at the lower tested concentration. Additionally, no resistance development, indicated by a delayed spheroid outgrowth, was observed over the whole time of treatment (20 to 40 days, depending on the size of the untreated spheroids). In the less sensitive SKMel30 cell line, a complete control of spheroid growth could be achieved by higher PMD-026 concentrations (Fig. 3B; Suppl. Figure S3).

Moreover, RSK inhibition also markedly impaired the anchorage-independent growth of melanoma cells in soft agar, which serves as an in vitro correlate for the tumorigenicity of transformed cells. A reduction in the colony formation was detectable for both established melanoma cell lines (Fig. 3C; Suppl. Figure S4A) and patient-derived short-term cultures of different mutational subgroups (Fig. 3D) upon a 10-d treatment with PMD-026. This could be confirmed with BI-D1870 as a second, RSK-specific inhibitor (Suppl. Figure S4B), and was also reflected in a decreased number of viable cells in the soft agar after PMD-026 treatment as assessed with an AlamarBlue-based cell viability assay (Suppl. Figure S4C, D). Consistent with the retained effect of RSK inhibitors on the cell cycle of MAPK pathway inhibitor-resistant melanoma cell lines (Suppl. Figure S2B), PMD-026 also impaired the anchorage-independent growth of these cells either alone or in combination with the MAPK inhibitors (Suppl. Figure S4E).

Based on the profound effect of RSK inhibition in suppressing tumor growth in vitro, its in vivo efficacy was assessed investigating a BRAF^Mut^ (451LU) and an NF-1^LOF^ (MeWo) subcutaneous melanoma xenograft model. In both model systems, treatment with the orally bioavailable RSK inhibitor PMD-026 significantly reduced the tumor growth in the immunocompromised mice (Fig. 3E, F; Suppl. Figure S5A, B). This went along with decreased phosphorylation of the RSK target YB-1 in the tumors (Suppl. Figure S5C, D). Of note, addition of the RSK inhibitor to a sub-effective concentration of the BRAF^V600E/K^ inhibitor vemurafenib restored tumor control in the BRAF^Mut^ melanoma xenograft model (Fig. 3E; Suppl. Figure S5C). These data suggest a therapeutic usefulness of the RSK inhibitor PMD-026 to target MAPK pathway-hyperactivated melanoma cells irrespective of their mutational subgroup and either as monotherapy or in combination with MAPK pathway inhibitors.

### Melanoma cell differentiation is restored by RSK inhibitors

**Fig. 4:**
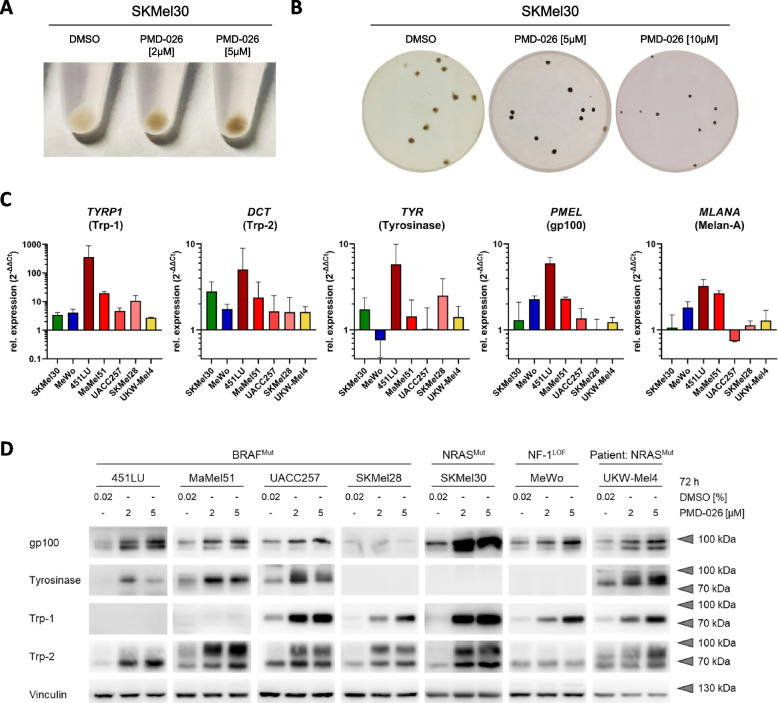
RSK inhibition promotes pigmentation and the expression of melanocyte differentiation antigens in melanoma cells. **A**,** B** Photographs of SKMel30 (NRAS^Mut^) melanoma cells (A: cell pellet after 72 h two-dimensional culture; B: 10 d spheroid culture on a 24-well plate) treated with increasing doses of PMD-026. **C** Real-time qPCR analysis for melanocyte differentiation antigen (MDA) transcript expression in melanoma cell lines of different mutational subgroups (NRAS^Mut^: green, NF-1^LOF^: blue, BRAF^Mut^: red) and a patient-derived short-term culture (yellow) after RSK inhibition (PMD-026 [5 µM], 72 h). *RPLP0* and *POLR2A* (RNA Polymerase II) were used as reference genes. Fold change induction by RSK inhibition was visualized by normalization to the respective solvent-treated control cells (mean ± SD; N ≥ 2 with *n* = 2). **D** Western blot analysis of MDAs in whole cell protein lysates after treatment with PMD-026 or its solvent control DMSO for 72 h. Vinculin served as loading control

Intriguingly, the functional effects of RSK inhibition on melanoma cells were not limited to the control of cell growth and survival, but also seemed to extend to the regulation of melanoma cell differentiation. This was evident in the NRAS^Mut^ cell line SKMel30, which appeared almost unpigmented under normal two- and three-dimensional growth conditions but visibly darkened after treatment with PMD-026 (Fig. 4A, B). In line with this, on a molecular level, RSK inhibitor treatment enhanced the mRNA and protein expression of various melanocyte differentiation antigens (MDAs) involved in melanin synthesis (Trp-1, Trp-2, Tyrosinase) and melanosome biogenesis (gp100, Melan-A). This was observed in established melanoma cell lines of all mutational subgroups as well as in patient-derived short-term cultures (Fig. 4C, D; Suppl. Figure S6A, B; Suppl. Table S9, S10).

A similar re-induction of MDA expression was achieved by MAPK pathway inhibition – with a MEK inhibitor in all cell lines or with a BRAF^V600E/K^ inhibitor in BRAF^Mut^ cell lines – suggesting that RSK serves as a downstream mediator of MAPK pathway-controlled melanoma cell dedifferentiation (Suppl. Figure S6C; Suppl. Table S11).

### RSK inhibition enhances the immunogenicity of melanoma cells

**Fig. 5:**
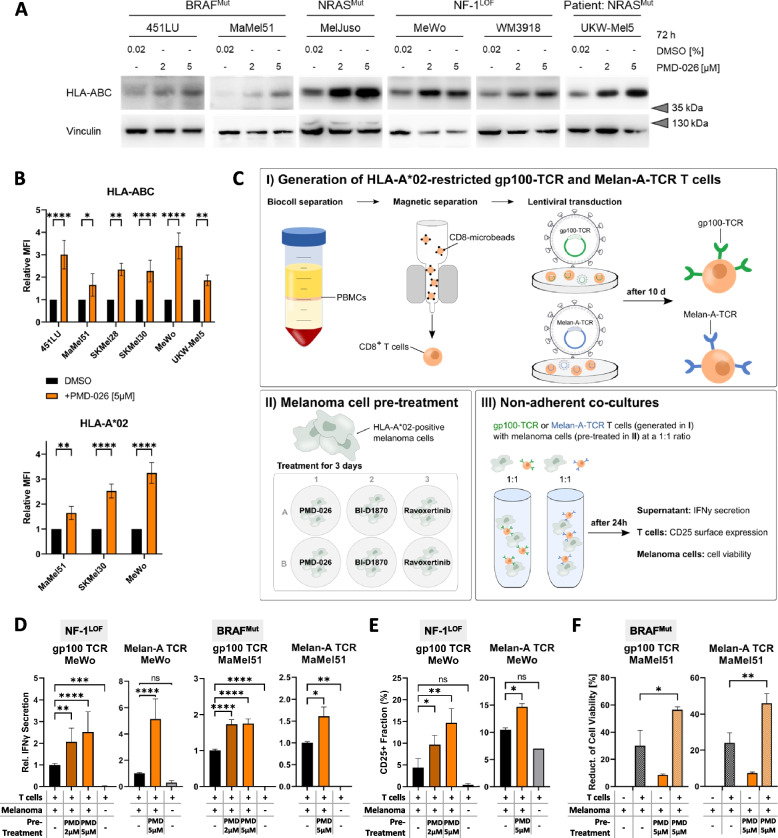
Surface MHC class I expression and immunogenicity of melanoma cells is augmented by RSK inhibition. **A**, **B** MHC class I protein levels and their localization at the cell surface of melanoma cells following RSK inhibitor treatment for 72 h. Immunoblot analysis was performed on whole cell protein lysates using Vinculin as loading control (**A**). Cell surface staining was conducted with FITC-coupled HLA-ABC- or APC-coupled HLA-A*02-specific antibodies, quantified by flow cytometry (mean fluorescence intensity, MFI) and normalized to the respective solvent control (mean ± SD, N ≥ 2) (**B**). Significance was determined by unpaired t-tests and subsequent Holm-Šídák’s multiple comparisons test. **C** Schematic workflow for non-adherent co-culture experiments with HLA-A*02-restricted gp100-TCR or Melan-A-TCR T cells and signaling pathway inhibitor pre-treated melanoma cells. **D**, **E** T-cell activation assessed by IFNγ secretion (**D**) and CD25 surface staining of gp100- or Melan-A-specific T cells (**E**) after 24 h co-culture of T cells with RSK inhibitor pre-treated melanoma cells (PMD-026, 72 h). Basal T-cell activation was assessed using the respective T cells without melanoma cell co-culture. Significance was assessed by one-way ANOVA with Dunnett’s correction for multiple comparison (mean ± SD; N ≥ 2). **F** AnnexinV-/ 7-AAD-based cell death staining of the RSK inhibitor pre-treated melanoma cells with and without 24 h co-culture with gp100- or Melan-A-specific T cells. The reduction of viable cell fraction (AnnexinV-negative / 7-AAD-negative population) compared to the control-treated melanoma cells without T-cell co-culture is indicated and significance was evaluated by two-way ANOVA with subsequent Bonferroni’s multiple comparisons test (mean ± SD; N ≥ 2)

Cancer cells often evade immune detection and destruction not only by downregulating tumor-specific antigens but also by disrupting the antigen-presenting machinery. Therefore, we further evaluated a potential effect of RSK inhibitors on the latter by assessing MHC class I protein expression. Both total levels of HLA-ABC as well as its localization to the cell surface was visibly enhanced by PMD-026 treatment throughout the distinct melanoma subgroups with MAPK pathway hyperactivation (Fig. 5A, B; Suppl. Figure S7A; Suppl. Table S12). These findings were corroborated by a simultaneous increase in surface expression of the specific MHC class I allele HLA-A*02 upon RSK inhibition in the applicable HLA-A*02-positive cell lines (Fig. 5B; Suppl. Figure S7B). Moreover, similar effects were achieved with the second RSK-specific inhibitor BI-D1870 as well as the ERK1/2 inhibitor ravoxertinib, which blocks RSK activation directly upstream (Suppl. Figure S7C, D). Interestingly, the potency of PMD-026 in upregulation of surface MHC class I seemed to be inversely correlated with the basal levels of HLA-ABC surface expression in the respective melanoma cell lines, with a strongest induction in 451LU und MeWo (Fig. 5B), which exhibit low MHC class I surface expression at baseline (Suppl. Figure S7E).

In order to assess if the combination of restored MDA expression and increased surface HLA-ABC upon RSK inhibition translates into an improved recognition by and activation of the immune system, primary human cytotoxic T cells were transduced with HLA-A*02-restricted, MDA-specific TCRs (gp100-TCR, Melan-A-TCR) and co-cultured with HLA-A*02-positive melanoma cell lines (Fig. 5C). Pre-treatment of the NF-1^LOF^ melanoma cell line MeWo and the BRAF^Mut^ MaMel51 with the RSK inhibitor PMD-026 could significantly enhance the activation of both gp100- and Melan-A-specific T cells as quantified by an increased IFNγ secretion (Fig. 5D). The specificity of this effect could be confirmed with the alternative RSK inhibitor BI-D1870, as well as with the ERK1/2 inhibitor ravoxertinib (Suppl. Figure S7F). In addition, increased surface CD25 expression demonstrated enhanced T-cell activation (Fig. 5E). Most importantly, going along with an increased activation of gp100- and Melan-A-specific T lymphocytes, the co-cultured melanoma cells were more efficiently eliminated by the MDA-TCR transduced T cells. This was reflected by an increased reduction of the viable tumor cell fraction in the co-cultures after RSK inhibitor pre-treatment (Fig. 5F).

Based on these findings, targeting p90 ribosomal S6 kinases might represent a promising therapeutic strategy for melanoma with hyperactivated MAPK pathway, since it not only directly affects melanoma cell viability, but also simultaneously restores tumor cell differentiation and immunogenicity, thus potentially boosting the anti-cancer immune responses in patients.

## Discussion

Recently, several pharmacological inhibitors of the MAPK pathway, targeting mutated BRAF (e.g. vemurafenib, dabrafenib, encorafenib) or MEK (e.g. cobimetinib, trametinib, binimetinib), have been developed and found their way into clinical application. However, despite initial responses of BRAF^Mut^ subtype melanomas to these inhibitors, their effects are frequently not durable and resistance develops within the first year of treatment in approximately half of the patients [9, 10]. Reactivation of MAPK signaling is a recurrent mechanism of resistance following BRAF and MEK inhibition [9, 11]. Consequently, current research has focused on inhibition of the MAPK signaling pathway further downstream. Indeed, inhibitors of ERK1/2 have already shown to efficiently inhibit MAPK signaling and proliferation of melanoma cells and their usefulness has been further evaluated in clinical trials (e.g. NCT01781429, NCT01875705) [24, 25]. In line with the ubiquitous involvement of the MAPK pathway in various essential cellular processes, however, ERK1/2 inhibitors have been shown to exhibit a considerable toxicity profile [26]. Therefore, attention has turned to the identification of novel potential target structures among the downstream effectors of MAPK signaling with a crucial, but ideally exclusive role in cancer cell biology. This should enable efficient anti-tumor activity while minimizing the adverse effects associated with their inhibition.

Considering that the RSK family is directly activated by ERK1/2 and regulates important tumorigenic processes in a variety of cancers [17], it represents such a potential target. Indeed, BRAF-mutated melanoma cells can be effectively targeted by RSK inhibition and its family members RSK1 and RSK2 have been reported to enhance melanoma cell survival under chemotherapy [18, 27, 28]. Unfortunately, until recently, a poor pharmacokinetic profile of the so-far available RSK inhibitors precluded their in vivo application [29, 30]. This limitation could now be finally overcome by the novel first-in-class RSK inhibitor PMD-026 with high specificity and improved oral bioavailability [31]. Both preclinical studies and a Phase I clinical trial in metastatic breast cancer (Clinical trial information: NCT04115306) demonstrated a good safety profile of PMD-026 and – in contrast to MAPK pathway inhibitors – no apparent cardiotoxicity, ocular toxicity or neutropenia in mice and dogs [31–33]. Most importantly, the RSK inhibitor was highly effective in targeting triple-negative breast cancer and prostate carcinoma not only in vitro but also in a pre-clinical in vivo setting [31, 32, 34] and showed initial signs of efficacy in metastatic breast cancer in a Phase I clinical trial [33].

Here we can show for the first time that the efficacy of PMD-026 and RSK inhibition in general extends to malignant melanoma cells with MAPK pathway hyperactivation, including not only the BRAF^Mut^ subgroup irrespective of the response to MAPK pathway inhibitors but also melanomas with RAS^Mut^ or NF-1^LOF^, for which no effective targeted treatment options have been yet available. Similar to the reports in breast and prostate cancer, where PMD-026 efficiently induced apoptosis in the tumor cells [31, 32, 34], RSK inhibition potently enhanced the sub-G1 cell cycle fraction as well as the protein levels of the pro-apoptotic Bim in the majority of melanoma cell lines. This is in agreement with the already described implication of RSK in apoptosis suppression, for example by inactivation of pro-apoptotic proteins such as Bim, Bad or the death-associated protein kinase 1 (DAPK1) [35–37]. More specifically, active RSK signaling induces the proteasomal degradation of Bim by its phosphorylation at S93/S94/S98 [37]. This explains the subsequent stabilization of Bim following RSK inhibition in addition to the marked reduction in phosphorylation of the well-described RSK targets YB-1 and SRF [18, 38, 39].

Beyond two-dimensional cell cultures, chronic RSK inhibitor treatment could achieve effective suppression of melanoma growth also in different three-dimensional in vitro model systems simulating more physiological growth conditions. These results were based both on established melanoma cell lines and on patient-derived short-term cultures. Most importantly, we can show here for the first time that the efficacy of RSK inhibitors in targeting malignant melanoma also prevails in an in vivo setting.

Intriguingly, the beneficial effects of RSK inhibition in melanoma cells seems not to be limited to the direct tumor growth control, but also extends to the restoration of their differentiation and immunogenicity. So far, a considerable fraction of melanoma patients shows intrinsic resistance to immunotherapeutic regimens [14, 15]. Although endogenous T cell responses against MDAs have been observed and provided the basis for adoptive T cell therapies (ACT) [40, 41], previous reports described a reversible loss of MDA expression leading to immune evasion and ACT resistance [42–44]. In this study, we revealed an enhanced activation of both gp100- and Melan-A-specific T cells as well as more efficient tumor cell killing following RSK inhibitor pre-treatment. This went along with an increased expression of melanocyte differentiation antigens and surface MHC class I after RSK inhibition. Since this is well in line with previous studies attesting similar effects to MAPK pathway inhibition with BRAF and MEK inhibitors [45–48], RSK seems to be an important mediator of the MAPK pathway induced immune evasive tumor state. Consequently, RSK inhibition could improve endogenous and adoptive T cell responses directed against melanocytic differentiation antigens and promote immunotherapy responsiveness in melanoma patients.

## Conclusions

In conclusion, RSK inhibitors may represent a novel therapeutic tool for malignant melanomas benefiting from their general usefulness in melanomas with dysregulated MAPK signaling, a tolerable toxicity profile as well as from their anti-tumor efficacy on two levels: first, a direct effect on proliferation and survival; and second, an enhanced immunogenicity of the tumor cells. This could not only enable melanoma cell eradication by the patient’s immune system but also increase the efficacy of immunotherapeutic strategies offering the prospect of long-lived clinical responses.

## Supplementary Information

**Additional file 1: Suppl. Figure S1.** Specific small molecule inhibitors suppress active RSK signaling in MAPK pathway hyperactivated melanoma cell lines.

**Additional file 2: Suppl. Figure S2.** PMD-026 increases the sub-G1 fraction in MAPK pathway inhibitor-resistant melanoma cells.

**Additional file 3: Suppl. Figure S3.** RSK inhibition attenuates spheroid growth of melanoma cells with MAPK pathway hyperactivation.

**Additional file 4: Suppl. Figure S4.** Anchorage-independent growth and colony formation of melanoma cell lines is suppressed by RSK inhibitors.

**Additional file 5: Suppl. Figure S5.** PMD-026 effectively inhibits RSK activity in melanoma cells in vivo.

**Additional file 6: Suppl. Figure S6.** MAPK/RSK inhibitors augment expression of pigmentation antigens.

**Additional file 7: Suppl. Figure S7.** Inhibition of the MAPK/RSK signaling axis increases surface MHC class I expression and melanoma cell immunogenicity.

**Additional file 8: Suppl. Table S1.** Established Melanoma Cell Lines and Patient-Derived Short-Term Cultures with the Respective MAPK Pathway Mutation. **Suppl. Table S2.** Oligonucleotide Primers for Real-Time Quantitative PCR Analysis. **Suppl. Table S3.** Primary Antibodies for Western Blot (WB), FACS and Immunohistochemical (IHC) Analyses. **Suppl. Table S4.** Densitometric Analysis of Immunoblots in Fig. 1A and Suppl. Figure S1A. **Suppl. Table S5.** Densitometric Analysis of Immunoblots in Fig. 1B and Suppl. Figure S1B. **Suppl. Table S6.** Densitometric Analysis of Immunoblots in Suppl. Figure S1C. **Suppl. Table S7.** Densitometric Analysis of Immunoblots in Figure 1C and Suppl. Figure S1E. **Suppl. Table S8.** Densitometric Analysis of Immunoblots in Suppl. Figure S1D. **Suppl. Table S9.** Densitometric Analysis of Immunoblots in Fig. 4D. **Suppl. Table S10.** Densitometric Analysis of Immunoblots in Suppl. Figure S6B. **Suppl. Table S11.** Densitometric Analysis of Immunoblots in Suppl. Figure S6C. **Suppl. Table S12.** Densitometric Analysis of Immunoblots in Fig. 5A.

 

**Additional file 9:** Supplementary Figure Legends.

## Data Availability

All data are available in the published article or the supplementary information files.

## Abbreviations

7-AAD: 7-Amino-Actinomycin
ACT: Adoptive T cell therapies
APC: Allophycocyanin
BRAF^Mut^: BRAF-mutated
d: Day
DAPK1: Death-associated protein kinase 1
DMSO: Dimethyl sulfoxide
FCS: Fetal calf serum
FITC: Fluorescein isothiocyanate
h: Hours
HLA-A*02: Human leukocyte antigen A*02
IFNγ: Interferon-γ
MHC: Major histocompatibility complex
MFI: Mean fluorescence intensity
MDAs: Melanocyte differentiation antigens
MAPK: Mitogen-activated protein kinase
NF-1^LOF^: NF-1 loss-of-function
NSG™: NOD *scid* gamma
NHM: Normal human melanocytes
NRAS^Mut^: NRAS-mutated
RSK: p90 ribosomal S6 kinase
ppm: Parts per million
PBMCs: Peripheral blood mononuclear cells
PBS: Phosphate buffered saline
P^S102^: Phosphorylation at serine 102
P^S380^: Phosphorylation at serine 380
P^T359^: Phosphorylation at threonine 359
qPCR: Quantitative polymerase chain reaction
R: Resistance to the BRAF inhibitor
RR: Resistance to the BRAF and MEK inhibitor combination
TCRs: T-cell receptors
TCGA: The Cancer Genome Atlas
V: Volume
WT: Wild-type
YB-1: Y-box binding protein 1
