# Supplementary figures and images for: Inhibition of p90 ribosomal S6 kinases disrupts melanoma cell growth and immune evasion
Source: J Exp Clin Cancer Res. 2023 Jul 19;42:175. doi: 10.1186/s13046-023-02755-5 (PMC10354913; doi:10.1186/s13046-023-02755-5)

Suppl. Figure 1

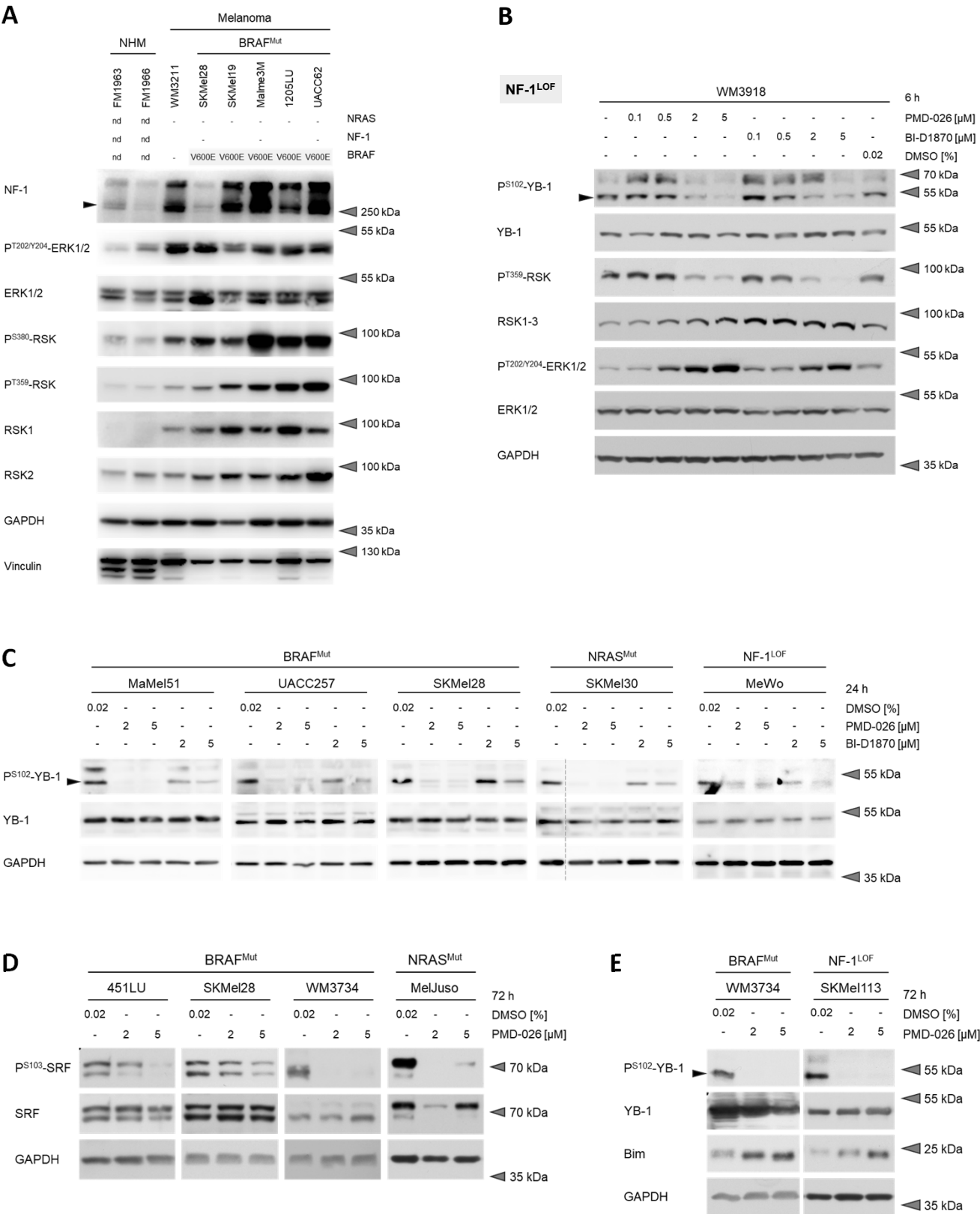

Supplement: Supplementary file 1 — Additional file 1: Suppl. Figure S1. Specific small molecule inhibitors suppress active RSK signaling in MAPK pathway hyperactivated melanoma cell lines. [file 13046_2023_2755_MOESM1_ESM.pdf]

Suppl. Figure 2

A

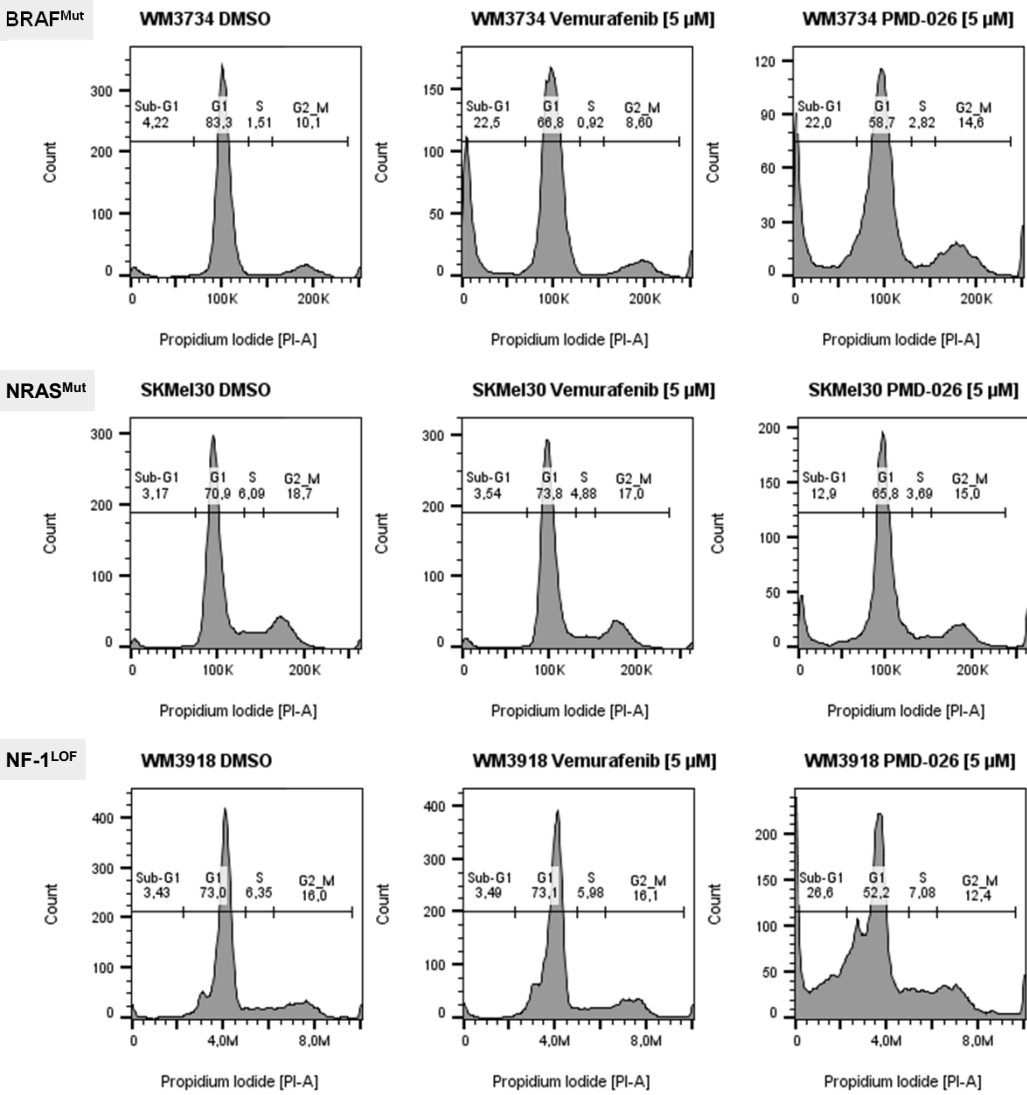

B

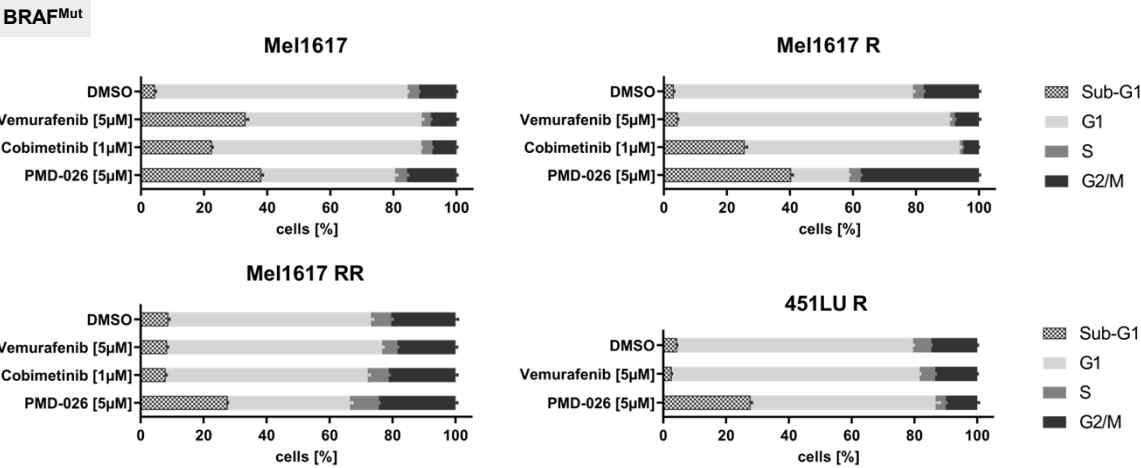

Supplement: Supplementary file 2 — Additional file 2: Suppl. Figure S2. PMD-026 increases the sub-G1 fraction in MAPK pathway inhibitor-resistant melanoma cells. [file 13046_2023_2755_MOESM2_ESM.pdf]

Suppl. Figure 4

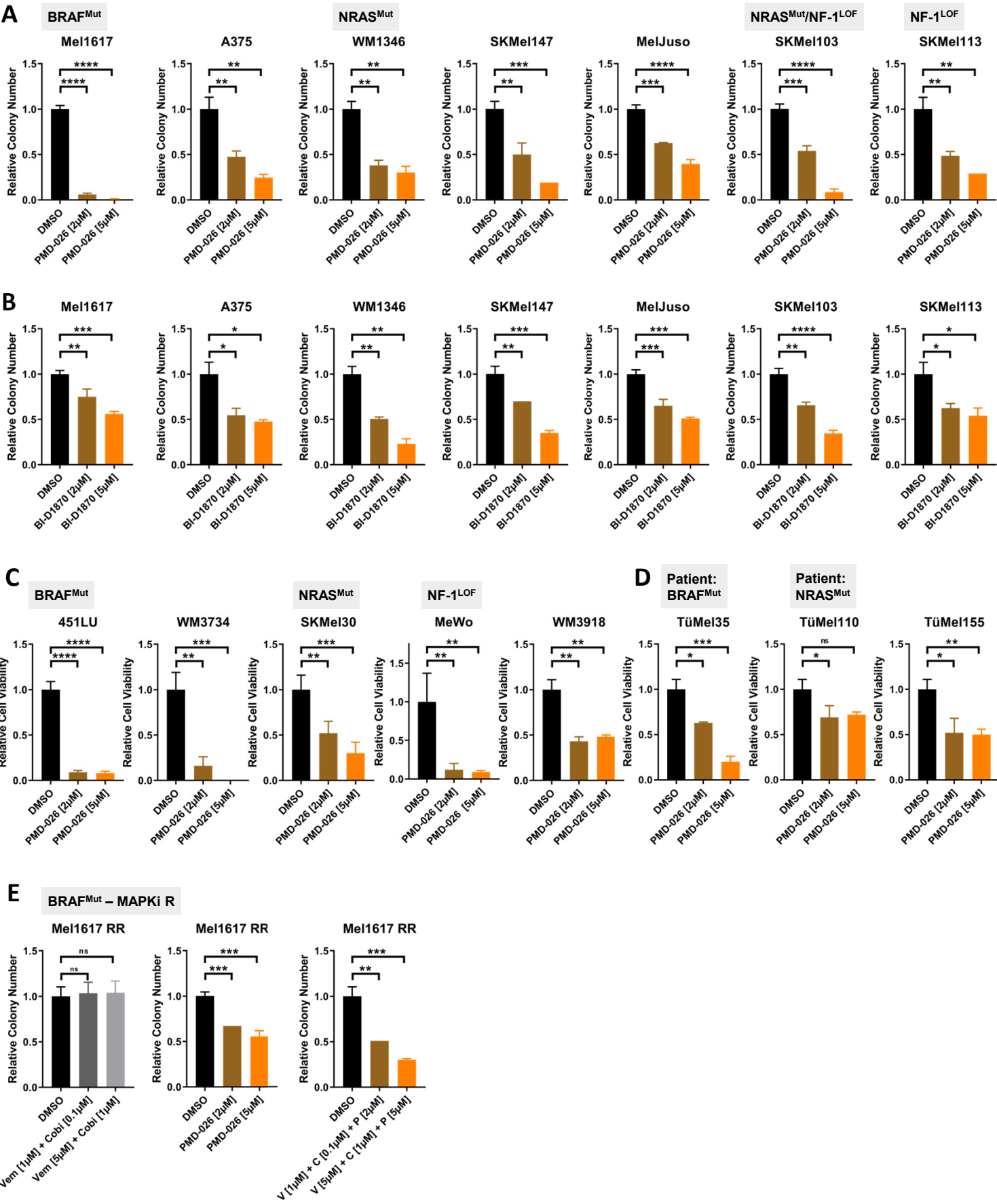

Supplement: Supplementary file 4 — Additional file 4: Suppl. Figure S4. Anchorage-independent growth and colony formation of melanoma cell lines is suppressed by RSK inhibitors. [file 13046_2023_2755_MOESM4_ESM.pdf]

Suppl. Figure 6

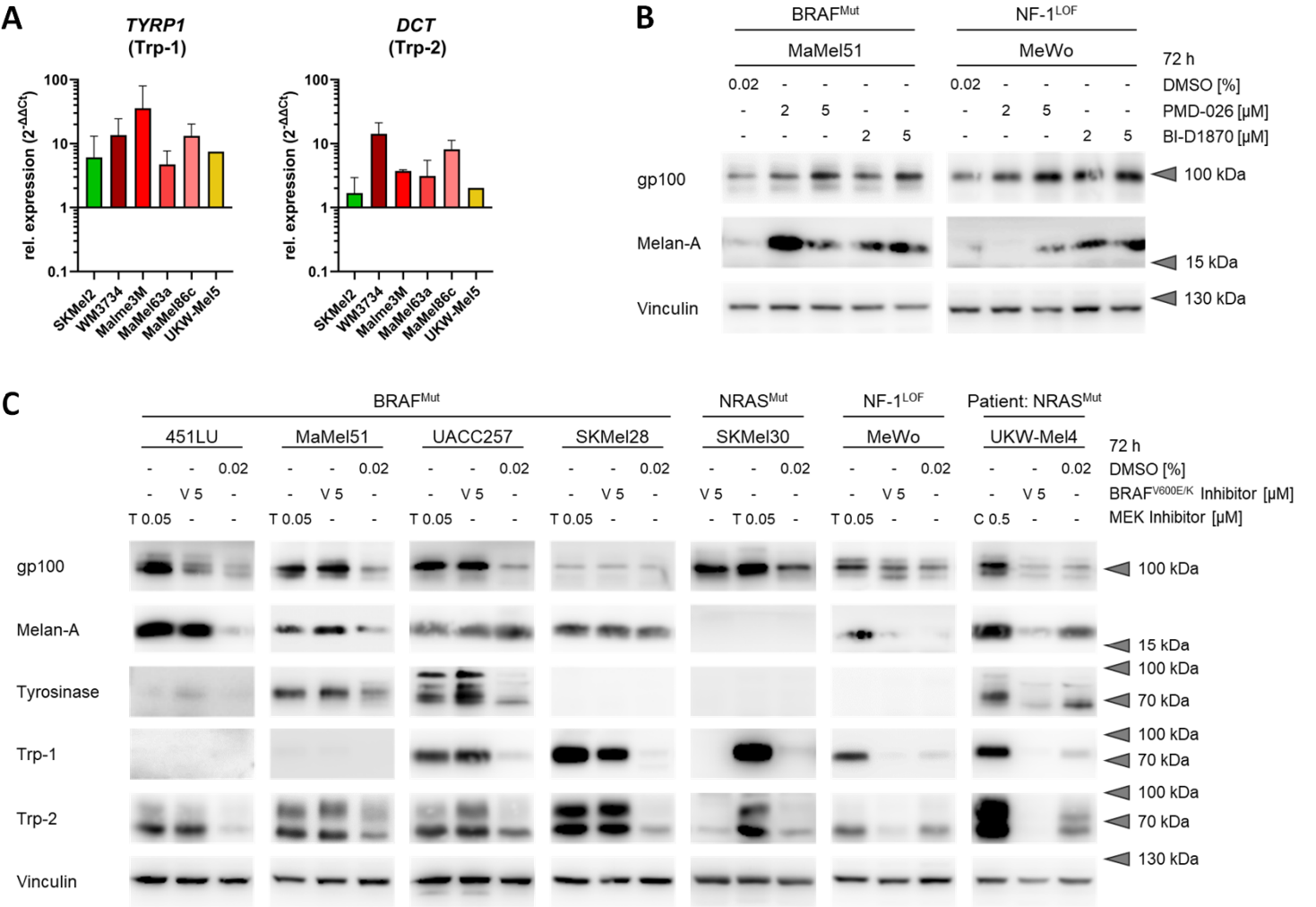

Supplement: Supplementary file 6 — Additional file 6: Suppl. Figure S6. MAPK/RSK inhibitors augment expression of pigmentation antigens. [file 13046_2023_2755_MOESM6_ESM.pdf]

Suppl. Figure 7

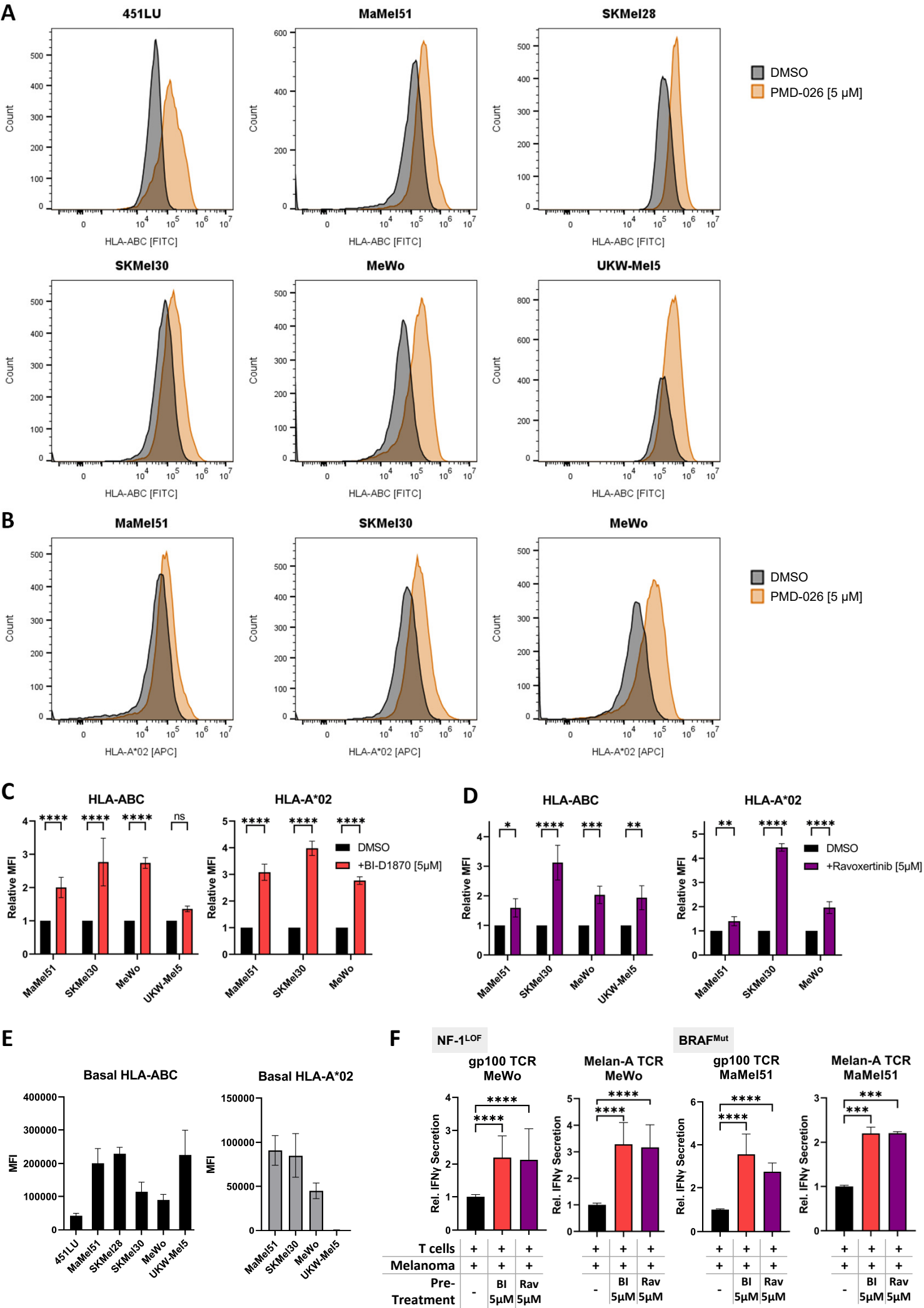

Supplement: Supplementary file 7 — Additional file 7: Suppl. Figure S7. Inhibition of the MAPK/RSK signaling axis increases surface MHC class I expression and melanoma cell immunogenicity. [file 13046_2023_2755_MOESM7_ESM.pdf]
