# Supplementary material for: Inhibition of p90 ribosomal S6 kinases disrupts melanoma cell growth and immune evasion
Source: J Exp Clin Cancer Res. 2023 Jul 19;42:175. doi: 10.1186/s13046-023-02755-5 (PMC10354913; doi:10.1186/s13046-023-02755-5)

Suppl. Figure 3

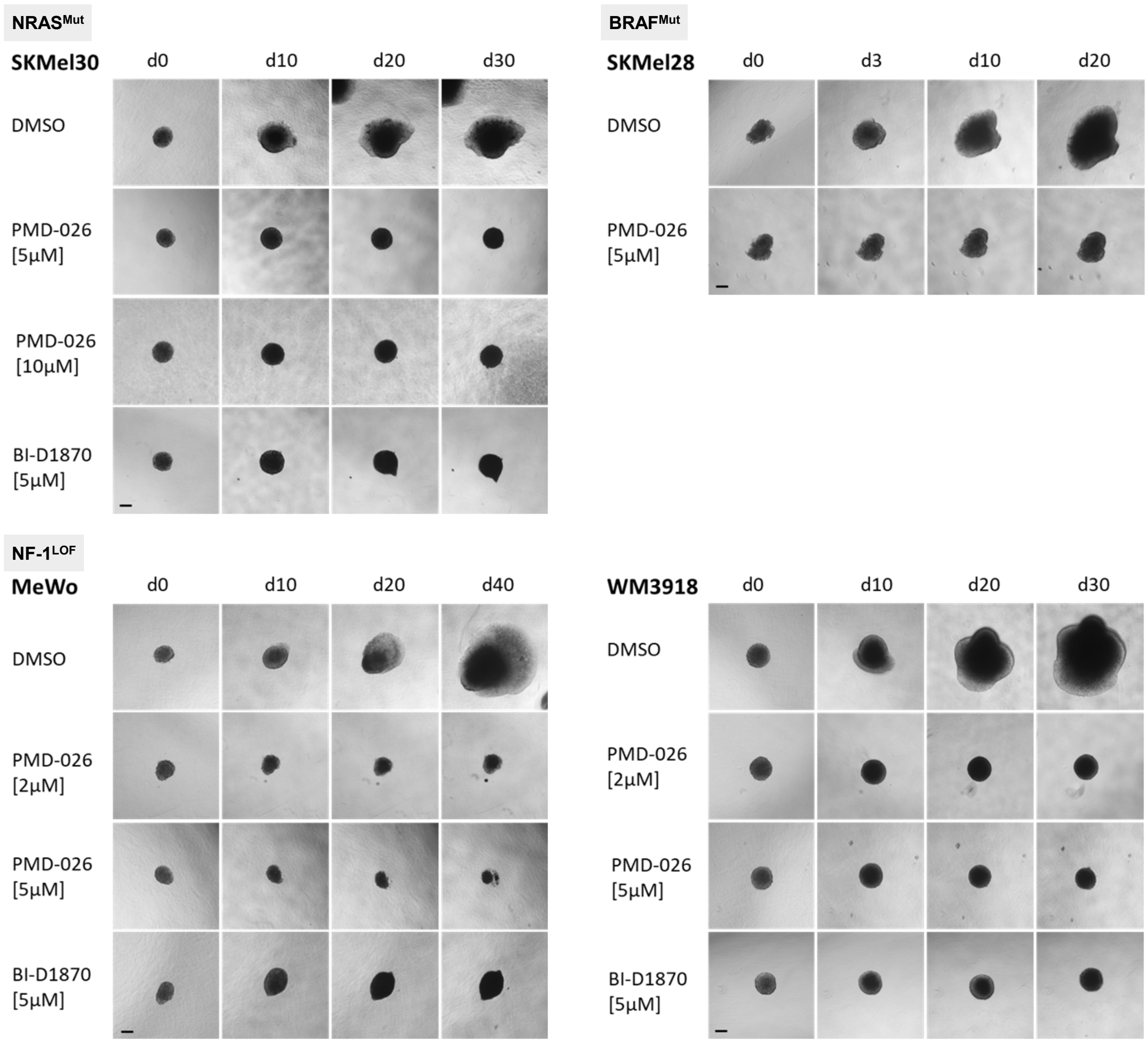

Supplement: Supplementary file 3 — Additional file 3: Suppl. Figure S3. RSK inhibition attenuates spheroid growth of melanoma cells with MAPK pathway hyperactivation. [file 13046_2023_2755_MOESM3_ESM.pdf]
