# Supplementary material for: Inhibition of p90 ribosomal S6 kinases disrupts melanoma cell growth and immune evasion
Source: J Exp Clin Cancer Res. 2023 Jul 19;42:175. doi: 10.1186/s13046-023-02755-5 (PMC10354913; doi:10.1186/s13046-023-02755-5)

Suppl. Figure 5

A **BRAF^{Mut} Xenograft : 451LU**

Therapy Group	n	Median Age	Sex M	Sex F	Mean Weight	Mean Tumour Volume at Therapy Start
**Vehicle**	7	90 d	3	4	26 g	34.7 mm^3
**PMD-026**	7	90 d	4	3	27 g	28.7 mm^3
**Vemurafenib + Vehicle**	6	90 d	3	3	27 g	34.7 mm^3
**Vemurafenib + PMD-026**	6	90 d	3	3	26 g	27.1 mm^3

B **NF-1^{LOF} Xenograft : MeWo**

Therapy Group	n	Median Age	Sex M	Sex F	Mean Weight	Mean Tumour Volume at Therapy Start
**Vehicle**	7	132 d	5	2	30.9 g	58.0 mm^3
**PMD-026**	7	122 d	6	1	30.1 g	60.1 mm^3

C

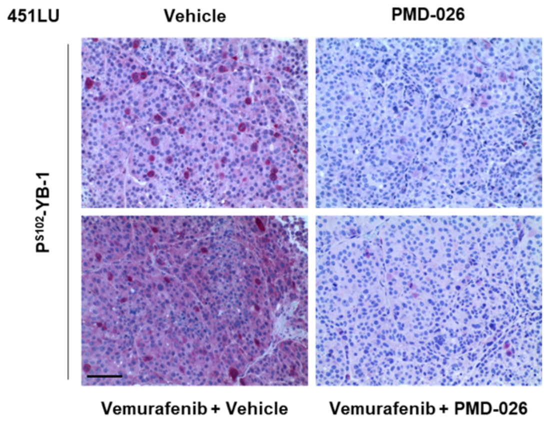

D

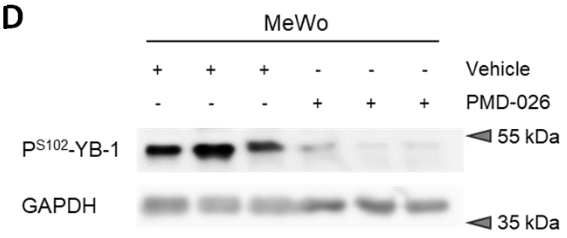

Supplement: Supplementary file 5 — Additional file 5: Suppl. Figure S5. PMD-026 effectively inhibits RSK activity in melanoma cells in vivo. [file 13046_2023_2755_MOESM5_ESM.pdf]
