# Supplementary material for: Inhibition of p90 ribosomal S6 kinases disrupts melanoma cell growth and immune evasion
Source: J Exp Clin Cancer Res. 2023 Jul 19;42:175. doi: 10.1186/s13046-023-02755-5 (PMC10354913; doi:10.1186/s13046-023-02755-5)
Supplement: Supplementary file 9 — Additional file 9: Supplementary Figure Legends. [file 13046_2023_2755_MOESM9_ESM.docx]

# Supplementary Figure Legends

## **Supplementary Figure S1:** Specific small molecule inhibitors suppress active RSK signaling in MAPK pathway hyperactivated melanoma cell lines.

**(A)** Immunoblot analysis of MAPK/RSK pathway activity in whole cell protein lysates of BRAF-mutated melanoma cells (BRAF^Mut^) and of benign control cells (NHM). GAPDH and Vinculin served as loading controls. **(B-E)** Western blot analysis of RSK target phosphorylation and expression in whole cell protein lysates of WM3918 (NF-1^LOF^, B) or a panel of melanoma cell lines with constitutive MAPK pathway activity (BRAF^Mut^, NRAS^Mut^, NF-1^LOF^, C-E) after treatment with RSK inhibitors (PMD-026, BI-D1870) or DMSO as solvent control. A dashed line marks image splicing to leave out irrelevant lanes in between. GAPDH was used as loading control.

## **Supplementary Figure S2:** PMD-026 increases the sub-G1 fraction in MAPK pathway inhibitor-resistant melanoma cells.

Flow cytometric cell cycle analyses of melanoma cells following a 72-h treatment with PMD-026, the BRAF^V600E/K^ inhibitor vemurafenib or the MEK inhibitor cobimetinib for 3 d (n = 3; mean ± SD). **(A)** Representative cell cycle profiles and relative distribution of the respective cell cycle phases for BRAF^Mut^, NRAS^Mut^ and NF-1^LOF^ melanoma cell lines presented in Figure 2B. **(B)** Flow cytometric cell cycle analyses of BRAF^Mut^ melanoma cells with acquired resistance to the BRAF^V600E/K^ inhibitor vemurafenib (R) or to both BRAF^V600E/K^ and MEK inhibitors (RR).

## **Supplementary Figure S3:** RSK inhibition attenuates spheroid growth of melanoma cells with MAPK pathway hyperactivation.

Melanoma spheroid growth in soft agar under RSK inhibition. Representative microphotographs of the spheroids quantified in Figure 3A and B are shown (scale bar represents 200 µm).

## **Supplementary Figure S4:** Anchorage-independent growth and colony formation of melanoma cell lines is suppressed by RSK inhibitors.

**(A-E)** Anchorage-independent growth assays of melanoma cell lines from different mutational subgroups (A-C; BRAF^Mut^, NRAS^Mut^, NF-1^LOF^), of patient-derived short-term cultures (D) or of melanoma cells with acquired MAPKi resistance (E) treated with RSK (PMD-026 (P), BI-D1870) and/or MAPK inhibitors (vemurafenib (V), cobimetinib (C)) for 10 d. The number of colonies formed was counted (N = 2 with n = 5, mean ± SD; A, B, E) or the total number of viable cells measured by means of AlamarBlue cell viability assays (N = 2 with n = 4, mean ± SD; C, D). Values were normalized to the solvent-treated controls, respectively, and significance determined by one-way ANOVA with subsequent Tukey’s multiple comparisons test.

## **Supplementary Figure S5:** PMD-026 effectively inhibits RSK activity in melanoma cells *in vivo*.

Xenograft growth of melanoma cells in NSG mice under RSK inhibitor therapy. **(A, B)** Characteristics of the therapy groups with sex, median age and mean weight of mice as well as tumor volumes at therapy start (A: 451LU; B: MeWo). **(C)** P^S102^-YB-1 immunohistochemical staining of 451LU xenograft tissue from the different therapy groups. YB-1 S102 phosphorylation levels are shown in red (Fast Red substrate) with a hematoxylin counter staining. Scale bar represents 50 µm. **(D)** Immunoblot analysis of YB-1 S102 phosphorylation in MeWo xenografts lysates. GAPDH served as loading control. The respectively received *in vivo* therapy is indicated.

## **Supplementary Figure S6**: MAPK/RSK inhibitors augment expression of pigmentation antigens.

**(A)** Real-time qPCR analysis of RSK inhibition-induced (PMD-026 [5 µM], 72 h) MDA transcript expression in melanoma cells (NRAS^Mut^: green, BRAF^Mut^: red, patient-derived: yellow). *RPLP0* and *POLR2A* were used as reference genes and target gene expression normalized to the respective solvent-treated control cells (mean ± SD; N ≥ 2 with n = 2, except UKW-Mel5: N = 1). **(B, C)** Immunoblot analysis of MDA expression in melanoma whole cell protein lysates after treatment with RSK inhibitors (B), MAPK inhibitors (C; vemurafenib (V), trametinib (T), cobimetinib (C)) or the solvent control DMSO for 72 h. Vinculin served as loading control. Protein bands of the solvent controls in (C) are in part identical to the control bands in Figure 4D.

## **Supplementary Figure S7:** Inhibition of the MAPK/RSK signaling axis increases surface MHC class I expression and melanoma cell immunogenicity.

**(A-E)** FACS-based cell surface staining with FITC-coupled HLA-ABC- or APC-coupled HLA-A*02-specific antibodies. Representative histograms of melanoma cells stained for HLA-ABC (A) or HLA-A*02 surface expression (B) after PMD-026 treatment for 72 h are shown. MAPK/RSK pathway inhibitor induced (C: BI-D1870; D: ravoxertinib) or basal (E) expression was quantified as mean fluorescence intensities (MFI). Inhibitor treated samples were normalized to the respective solvent control (C, D; mean ± SD). Significance was calculated by unpaired t-tests with subsequent Holm-Šídáks’s multiple comparisons test (C, D; N ≥ 2). **(F)** ELISA-based quantification of IFNγ secretion after 24 h co-culture of gp100- or Melan‑A-specific T cells with RSK or ERK1/2 inhibitor pre-treated melanoma cells (BI-D1870 or ravoxertinib, 72 h). Significance was determined by one-way ANOVA with Dunnett’s correction for multiple comparison (mean ± SD; N ≥ 2).
